# Key steps to readjust a maternal, newborn, and child health program at the time of a major funding reduction: Lessons learned from the SHINE transition in Somalia

**DOI:** 10.7189/jogh.12.03056

**Published:** 2022-08-09

**Authors:** Mitsuaki Hirai, Sandrine Chopin, Silvia Gatscher, Penelope Campbell

**Affiliations:** 1 UNICEF Somalia, Health Section, Nairobi, Kenya; 2 UNICEF Somalia, Health Section, Mogadishu, Somalia

The COVID-19 pandemic has caused a great deal of mortality and morbidity worldwide with 431.5 million cases and 5.9 million deaths as of February 25, 2022 [1]. Due to major economic turmoil, an additional 110 to 150 million people were estimated to live in extreme poverty by the end of 2021 [2]. It has also affected the flow of development and humanitarian funding from high-income countries to low- and middle-income countries. The amount of official development assistance (ODA) by the United Kingdom, for instance, was reduced from 0.7% of the Gross National Income (GNI) in 2020 to 0.5% in 2021 [3]. Under such circumstances, humanitarian and development programs may need to adjust their programmatic scope in accordance with available financial resources.

In Somalia, the United Nations Children’s Fund (UNICEF) implemented the Somali Health and Nutrition Programme (SHINE) from October 2019 to March 2022 as one of the three organizations to manage funding from the UK Government [4]. The main objective of SHINE was to provide maternal, newborn, and child health (MNCH) services and nutritional support to vulnerable populations across the country. Although SHINE was implemented as a development program, it also provided humanitarian assistance through COVID-19 response activities, such as the provision of personal protective equipment and infection prevention and control training to health facility staff. This program was expected to end in September 2021 initially, but it was extended up to March 2022.

The total SHINE program funding from the UK Government was reduced by 30% from GBP 20 million (approximately US$25.6 million) in 2020 to GBP 14 million (approximately US$19.18 million) in 2021, which compelled the SHINE program to revise its geographic and programmatic scope. UNICEF closely worked with the UK Government and the other fund managers to complete the SHINE program transition and identified many lessons to inform future program planning. The aim of this field note is 2-fold: 1) to summarize the practical steps that UNICEF followed to reduce the scope of a multi-year health program with reduced financial resources, and 2) to describe lessons learned from the SHINE program transition to inform how humanitarian and development programs may adapt to the COVID-19 induced economic environment.

## KEY STEPS FOR THE SHINE PROGRAM TRANSITION

The first step for the SHINE program transition was to reduce program coverage in phases with available financial resources. In early 2020, the UK Government communicated with three fund managing organizations – Mott MacDonald (Mott), Population Services International (PSI), and UNICEF – regarding the phased reduction of SHINE program funding in 2021. The UK Government also announced that UNICEF would be the only fund managing organization from July 2021 to complete this multi-year program in September 2021. Based on these announcements and available financial resources, UNICEF implemented SHINE program activities through 95 service delivery points (87 health facilities and eight mobile and outreach services) from October 2019 to June 2020, 86 service delivery points (78 health facilities and eight mobile and outreach services) from July 2020 to March 2021, and 50 service delivery points (49 health facilities and one mobile service) from April to June 2021.

**Figure:**
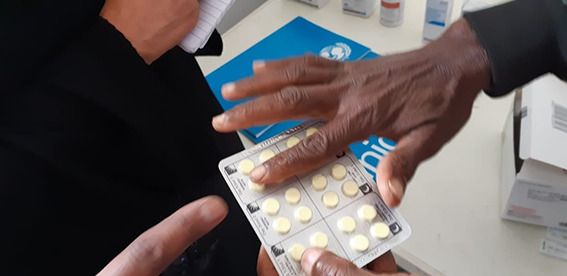
Photo: A SHINE-supported facility in Somalia. Source: UNICEF Somalia.

The second step was to consolidate SHINE-supported health facilities by three fund managers, review program data, financial resources, and supply availability, and determine the revised SHINE program coverage from July 2021. The SHINE program coverage was reviewed and adjusted in accordance with the following guiding principles: 1) achieving the optimal health outcomes possible with available funds; 2) standardizing program activities; 3) minimizing the risk of duplicative efforts with other donors and partners; 4) maintaining the operational presence in the current SHINE-supported districts; 5) keeping the existing partners; and 6) continuing the essential package of health services (EPHS) at health facilities; 7) maintaining basic emergency obstetric and newborn care (BEmONC) and comprehensive emergency obstetric and newborn care (CEmONC) in designated health facilities; and 8) providing the same level of program supervision as before.

Using these guiding principles, Ministries of Health, the UK Government, three fund managing organizations, and implementing partners reviewed the health service utilization data to assess the performance of health facilities in service provision, geographic locations of SHINE-supported health facilities, historical contexts of program locations, and available funding per implementing partner. A list of factors considered to identify priority health facilities for the updated SHINE program are presented in **Table 1**.

**Table 1:** Key factors considered to identify priority health facilities for the SHINE Program

Type of criteria	Key factors considered for health facility selection
Health service utilization	Number of outpatient department (OPD) visits, number of antenatal care (ANC) visits, number of deliveries with skilled birth attendance, number of immunizations (measles and pentavalent), number of health facility staff supported, type of health services available in each facility
Geography	Distance to SHINE-supported health facilities by patients, distance between SHINE-supported health facilities, accessibility from IDP camps, district size
Financial resources	Presence of other partners, other grants that may be available to support SHINE-supported health facilities, cost drivers

Facility-level data were retrieved from the District Health Information System 2 (DHIS2) for the period from January 2020 to March 2021. The criteria for identifying low-performing health facilities were the following: 1) less than 10 outpatient department (OPD) consultations per day for health centers and hospitals or 3 consultations per day for primary health units; 2) less than 3 vaccinations per day; 3) less than 3 antenatal care (ANC) consultations per day; or 4) less than 6 deliveries with skilled birth attendance per day. Additionally, UNICEF estimated the average number of consultations or services provided per health staff (average number of deliveries supported per nurse or midwife in health facilities). Based on the health service utilization analysis, geographic factors, and financial factors, UNICEF and implementing partners determined priority health facilities and retained them for the revised SHINE program. Overall, the total number of SHINE service delivery points decreased from 267 (260 health facilities and seven mobile and outreach services) by three fund managers in June 2021 to 192 (186 health facilities and six mobile and outreach services) by UNICEF from July 2021.

The third step was to prepare program documents (including key activities, performance indicators, and targets) with implementing partners. While the initial plan was to continue the SHINE program only up to September 2021, UNICEF prepared the program documents with the implementation period up to March 2022 in anticipation of the SHINE program extension. Each program document indicated that the program implementation beyond September 2021 was subject to the availability of funding. As the only fund manager, UNICEF activated program partnerships with six non-government organizations (NGOs) with this funding condition. The SHINE program was later extended up to March 2021 with some additional funding from the UK Government, and UNICEF also mobilized other financial resources to maintain the same condensed program level from July 2021 to March 2022.

## LESSONS LEARNED

The process of SHINE program transition generated several lessons. First, revising the scope of a large development program may require a great deal of time from donors, fund managers, and implementing partners. The SHINE program transition took nine-month-long coordination, data review, and program partnership development among key stakeholders to implement activities with a revised scope. When existing development programs need to modify the financial and programmatic scope due to reduced funding availability, adequate planning time may need to be allocated in preparation for the anticipated changes. Second, development programs with uncertain funding availability may set a flexible program implementation period. UNICEF activated program partnerships with implementing partners for nine months without a firm confirmation of additional funding from the UK Government. This approach contributed to minimizing the risk of program implementation gaps from the time-intensive partnership amendment or extension process. Future development programs may consider activating partnerships with implementing partners with a buffer implementation period with a note that the program continuation is subject to the availability of funding. Third, the sustainability of development programs remains in question in Somalia. The SHINE program transition highlighted that the cessation of funding may stop essential quality health service provision in many health facilities where the government budget cannot cover supply and personnel costs.

## CONCLUSIONS

This field note summarized the three major steps followed by UNICEF to revise the financial and programmatic scope of a major health program in Somalia and highlighted key lessons learned. In the era of reduced funding availability for development programs, allocating adequate time for the transition process, ensuring flexible program arrangements, and using data and contextual information to inform the decision-making process are vital. A key recommendation for the future is to consolidate available resources and implement multi-sectoral and multi-year interventions to respond to the needs of vulnerable populations on health, nutrition, water, sanitation, and hygiene (WASH) and other humanitarian assistance. By overcoming siloed approaches, development interventions may maintain a major impact even with reduced funding availability.
